# The effects of replacing fish meal or soy protein concentrate with wheat gluten on growth, whole-body composition, and the retention and apparent digestibility coefficient of amino acids in Japanese seabass (*Lateolabrax japonicus*)

**DOI:** 10.3389/fphys.2023.1220192

**Published:** 2023-06-15

**Authors:** Yuexing Zhang, Linghua Wang, Zhiyong Dong, Samwel Mugeni Changarawe, Liying Huang, Jinwei Hu, Trond Storebakken, Bo Shi

**Affiliations:** ^1^ National Engineering Research Center for Marine Aquaculture, Marine Science and Technology College, Zhejiang Ocean University, Zhoushan, Zhejiang, China; ^2^ Faculty of Bioscience, Department of Animal and Aquaculture Science, Norwegian University of Life Science, Ås, Norway; ^3^ Zhejiang Marine Fisheries Research Institute, Zhoushan, Zhejiang, China; ^4^ Dongguan Yihai Kerry Syral Starch Technology Co., Ltd., Dongguan, Guangdong, China

**Keywords:** wheat gluten, fish meal, soy protein concentrate, Japanese seabass, methionine requirement

## Abstract

The aim of this study was to evaluate the effects of replacing fish meal (FM) or soy protein concentrate (SPC) with wheat gluten on growth performance, feed utilization, and nutrient digestibility and retention in Japanese seabass (*Lateolabrax japonicus*). Seven isonitrogenous (441–456 g kg^−1^ crude protein) and isocaloric (21.5–22.0 MJ kg^−1^ gross energy) diets were produced to replace 0%, 33.3%, 66.7% and 100% of FM or SPC with a mixture of wheat gluten, wheat, and taurine (GWT, 77.5% wheat gluten, 20.5% wheat and 2.0% taurine). The gradual replacement of protein in FM with GWT had no significant effects on feed intake, whole-body composition, and the hepatosomatic and viscerosomatic indices, but resulted in a linear decrease in the weight gain rate, feed efficiency, and retention of nitrogen, energy, and essential amino acids (Arg, His, Ile, Leu, Lys, Met, Phe, Thr, and Val). The apparent digestibility of most essential amino acids (Cys, His, Leu, Lys, and Phe) and total amino acids increased linearly. Replacement protein in SPC with GWT had no significant effects on feed intake, growth, the feed conversion ratio, whole-body composition, and the hepatosomatic index, but resulted in a linear decrease in nitrogen, energy, and Met retention; the digestibility of Cys and Met increased linearly. Overall, wheat gluten is a more effective alternative for replacing protein in SPC than FM.

## 1 Introduction

The production of high-quality (LT) fish meal is highly standardized in terms of raw material freshness, separation, and the drying process. LT fishmeal has a high concentration of digestible protein and a balanced amino acid composition (Anderson et al., 1997). The lipid portion of LT fishmeal consists of triglycerides and phospholipids and high levels of n-3 fatty acids, which can provide essential fatty acids for fish (Olsen and Hasan, 2012). LT fishmeal is produced with presscake and stickwater, which contribute to the biological value of protein and the physical characteristics of extruded feed. However, the limited amount of fish meal and growing demand have led to higher prices (Tacon and Metian, 2008). Thus, it is necessary to find alternative protein sources to meet the protein requirement for fish growth in intensive aquaculture.

Wheat gluten is a proteinaceous material obtained from wheat, with a crude protein (CP) content of up to 80% (Apper-Bossard et al., 2013). It is a digestible binder in extruded feeds and has a protein digestibility close to 100% in the feed of Atlantic salmon (*Salmo salar*) and rainbow trout (*Oncorhynchus mykiss*) (Storebakken et al., 2000a; 2015a). In addition, it contains a relatively high concentration of sulfur-containing amino acids, including 1.8% Met and 2.6% Cys, which is considerably higher than in soy protein concentrate (Apper-Bossard et al., 2013). Wheat gluten is virtually devoid of antinutritional factors due to the low content of non-starch polysaccharides and other indigestible carbohydrates (Tusche et al., 2012). A study in salmon showed that wheat gluten can efficiently replace high-quality (LT) fish meal (Storebakken et al., 2015a). Another finding indicated that wheat gluten could improve intestinal health in salmon, which is probably related to the high glutamine content (35%–40% of CP) (Apper et al., 2016).

Soy protein concentrate (SPC) is a major protein source in fish. It contains a higher protein concentration than soybean meal, especially Arg, which can support the rapid growth and efficient feed conversion of fish (Storebakken, 2000b; Zhang et al., 2012b). Furthermore, antinutritional factors, such as protease inhibitors, lectins, soluble non-starch polysaccharides, and indigestible oligosaccharides, have been inactivated or removed (Storebakken, 2000b). However, the main challenge for SPC is phytate, which can be concentrated during the extraction process. Phytate has potentially negative effects on freshwater eutrophication due to its low availability. In warm-water fish, it is feasible to overcome this challenge by applying phytase in feed (Denstadli et al., 2007).

Japanese seabass *Lateolabrax japonicus*, known as black-spotted bass, is one of the most important aquaculture species in China. Studies on fish meal substitution in Japanese seabass have been widely reported. Juvenile Japanese seabass adapted to a 50% dietary soybean meal (IBW of 6.7 g) but required additional crystalline methionine supplementation (Zhang et al., 2016; Zhang et al., 2018). However, its tolerance to canola rapeseed meal is lower at less than 20% (Cheng et al., 2010). Men et al. (2014) found that replacing 60% fishmeal with corn gluten meal did not hinder the growth of Japanese seabass. Hu et al. (2013) evaluated the effects of different qualities of fish meal in larger Japanese seabass (IBW of 76 g), and the results indicated that prime steam-dried fishmeal was superior to standard steam-dried fishmeal in terms of feed intake and growth rate. Therefore, the aim of this study was to evaluate the effects of replacing LT fishmeal or SPC with wheat gluten in Japanese seabass, which could contribute to further understanding the potential use of wheat gluten as a considerable alternative protein source.

## 2 Materials and methods

### 2.1 Ingredients and diets

Seven isonitrogenous (441–456 g kg^-1^ crude protein) and isocaloric (21.5–22.0 MJ kg^-1^ gross energy) diets were formulated to compare the nutritional value and combination effects of wheat gluten, LT fishmeal, and SPC. The chemical compositions of wheat gluten, FM, and SPC are presented in Table 1. A mixture (GWT) consisting of 77.5% wheat gluten, 20.5% wheat, and 2.0% taurine was produced. The V0, VF1, VF2, and VF3 diets were formulated with GWT gradually replacing 0, 33.3, 66.7, and 50% FM, respectively. Similarly, GWT gradually replaced 33.3, 66.7, and 100% SPC to form the VS1, VS2, and VS3 diets, respectively (Table 2). Based on previous studies (Mai et al., 2006; Li et al., 2012; Hu et al., 2013), the V0 diet could meet the nutrient requirements of Japanese seabass. To maintain the same level of dietary essential amino acids (EAA), the VS1, VS2, and VS3 diets were supplemented with crystalline Lys, Arg, and Thr, and the VF1, VF2, and VF3 diets were supplemented with crystalline Lys, Arg, Thr, and Met. In addition, monocalcium phosphate was also added to the diets, and yttrium oxide (Y_2_O_3_) was used as an inert marker for digestibility assessment (Austreng et al., 2000; Zhang et al., 2006). The formulation, proximate compositions, and amino acid profiles of the diets are presented in Table 3, 4.

**TABLE 1:** Compositions of the three main ingredients in the experiment (dry matter basis).

Ingredient	Fish meal^a^	Wheat gluten^b^	Soy protein concentrate^c^	
Composition, kg^-1^				
Dry matter, g	936	946	925	
Crude protein, g	744	872	694	
Crude fat, g	117	44	47	
Starch, g	-	74	-	
Ash, g	122	10	58	
Gross energy, MJ	21.78	22.02	20.08	
Essential amino acids (EAAs), g (16 g N)^−1^				
Arg	5.28	3.43	7.46	
His	2.26	1.83	2.74	
Ile	4.07	3.54	4.77	
Leu	6.97	6.82	7.85	
Lys	7.40	1.65	6.43	
Met	2.67	2.02	1.33	
Phe	3.71	5.89	5.10	
Thr	3.98	2.67	4.00	
Trp	0.61	0.80	1.32	
Val	4.96	3.90	4.96	
Total EAAs	41.9	32.6	45.9	
Total non-essential AAs^d^	40.6	61.5	50.5	
Total AAs^d^	82.5	94.1	96.4	

^a^ Triple nine^®^, low-temperature dried fish meal, Esbjerg, Denmark.

^b^ AMYGLUTEN 110, Syral, Aalst, Belgium.

^c^ Yihai^®^.Wilpromil, Glodensea Grain and Oil Industry Co., Ltd, Wilmar, Qinhuangdao, China.

^d^ Tyr excluded.

**TABLE 2:** Inclusion rate of GWT, FM, and SPC in the experiment diets.

Diet	GWT, %	FM, %	SPC, %
V0	0	50	50
VF1	16.7	33.3	50
VF2	33.3	16.7	50
VF3	50	0	50
VS1	16.7	50	33.3
VS2	33.3	50	16.7
VS3	50	50	0

GWT, a mix of wheat gluten, wheat, and taurine; FM, fish meal; SPC, soy protein concentrate.

**TABLE 3:** Feed formulations and analyzed chemical compositions (dry matter basis).

Diets	V0	VF1	VF2	VF3	VS1	VS2	VS3
Ingredients, g kg^-1^							
GWT^a^	—	69.0	138.0	207.0	69.0	138.0	207.0
Fish meal^b^	200.0	133.0	66.0	—	200.0	200.0	200.0
Soy protein concentrate^c^	214.0	214.0	214.0	214.0	142.0	71.0	—
Soybean meal^d^	70.0	70.0	70.0	70.0	70.0	70.0	70.0
Peanut meal^d^	70.0	70.0	70.0	70.0	70.0	70.0	70.0
Krill meal^e^	50.0	50.0	50.0	50.0	50.0	50.0	50.0
Wheat flour^f^	266.4	250.4	234.6	217.8	262.9	259.1	255.4
Fish oil	84.0	89.0	94.0	99.0	84.0	84.0	84.0
Soy lecithin	20.0	20.0	20.0	20.0	20.0	20.0	20.0
Premix^g^	10.0	10.0	10.0	10.0	10.0	10.0	10.0
Mono calcium phosphate	14.0	17.0	20.0	23.0	14.5	14.5	14.5
Choline Cl	1.5	1.5	1.5	1.5	1.5	1.5	1.5
Y_2_O_3_	0.1	0.1	0.1	0.1	0.1	0.1	0.1
*L*-Lysine^h^	—	3.7	7.4	11.0	3.1	6.1	9.1
*DL*-Methionine^i^	—	0.4	0.8	1.2	—	—	—
*L-*Arginine^h^	—	1.1	2.1	3.1	2.1	4.2	6.2
*L-*Threonine^h^	—	0.8	1.5	2.3	0.8	1.5	2.2
Analyzed content, kg^-1^							
Dry matter, g	951	954	957	956	957	957	957
Crude Protein, g	441	447	444	449	447	451	456
Crude Fat, g	135	136	139	142	140	141	131
Ash, g	76	69	61	54	72	68	64
Gross Energy, MJ	21.5	21.6	21.6	22.0	21.5	21.7	21.8

^a^ Mixture of vital wheat gluten, wheat flour, and taurine (mixing ratio: 77.5%, 20.5%, and 2%).

^b^ Triple 9^®^. Low-temperature dried fish meal, Esbjerg, Denmark.

^c^ Yihai^®^. Wilpromil, Glodensea Grain and Oil Industry Co., Ltd, Wilmar, Qinhuangdao, China.

^d^ Fengyuan^®^. Glodensea Grain and Oil Industry Co., Ltd, Wilmar, Qinhuangdao, China.

^e^ QRILL™.Antarctic Krill Meal, Aker BioMarine, Oslo, Norway.

^f^ Bluekey^®^. Beijing Grain and Oil Industry Co., Ltd, Wilmar, Beijing, China.

^g^ Vitamin premix (mg kg^−1^ diet): vitamin A 20; vitamin B_1_ 12; vitamin B_2_ 10; vitamin B_6_ 15; vitamin B_12_ 8; niacinamide 100; ascorbic acid 1,000; calcium pantothenate 40; biotin 2; folic acid 10; vitamin E 400; vitamin K_3_ 20; vitamin D3 10; inositol 200; corn protein powder 150. Mineral premix (mg kg^−1^ diet): CuSO_4_ · 5H_2_O 10; FeSO_4_ · H_2_O 300; ZnSO_4_ · H_2_O 200; MnSO_4_ · H_2_O 100; KI (10%) 80; Na_2_SeO_3_ (10% Se) 67; CoCl_2_ · 6H_2_O (10% Co) 5; NaCl 100; zeolite 638. Vitamin premix: mineral premix = 2: 1.5.

^h^ *L-*Lysine HCL, *L-*Arginine Base, *L*-Threonine, 98% feed grade, Siwei Development Group Ltd., Hangzhou, China.

^i^ MetAMINO^®^ *DL*-Methionine, 99% feed grade, Evonik-Degussa Antwerpen N.V, Antwerpen, Belgium.

**TABLE 4:** Amino acid compositions of the experimental diets, g (16 g N)^−1^.

Diet	V0	VF1	VF2	VF3	VS1	VS2	VS3
Essential amino acids (EAAs)							
Arg	6.60	6.38	6.31	6.29	6.50	6.31	6.24
His	2.13	1.99	2.07	2.05	1.98	1.88	1.79
Ile	4.12	3.90	3.71	3.80	3.89	3.71	3.54
Leu	7.31	7.04	6.89	6.98	7.03	6.76	6.57
Lys	6.24	5.91	5.97	5.81	6.03	5.71	5.60
Met	1.58	1.53	1.39	1.42	1.72	1.75	1.80
Phe	4.72	4.61	4.84	4.84	4.57	4.43	4.39
Thr	3.90	3.74	3.64	3.64	3.79	3.66	3.60
Val	4.51	4.25	4.05	4.04	4.36	4.15	3.98
Total EAAs	41.1	39.4	38.9	38.9	39.9	38.4	37.5
Semi-EAAs							
Cys	0.91	1.03	1.08	1.24	1.05	1.10	1.14
Tyr	3.33	3.22	3.20	3.28	3.23	3.12	3.04
Total non-EAAs	52.9	53.4	54.6	57.4	53.3	53.5	54.5
Total AAs	94.0	92.8	93.5	96.3	93.2	91.9	92.0

### 2.2 Feed manufacturing and physical quality analysis

Diets were produced at the Feed Technology Laboratory of the Feed Research Institute, Chinese Academy of Agricultural Sciences in Beijing. All dry ingredients were ground in a hammer mill through a 0.18-mm screen, then mixed, preconditioned, and extruded in a twin-screw extruder (MY56X2A, Muyang, Jiangsu, China) with a 2.0 mm die plate (MY56A 12-03/02 XL 09 11). The goal of the extrusion process was to achieve a bulk density higher than 516 g L^−1^ prior to drying to facilitate the slow sinking of feed after drying and lipid coating. All extruded pellets were dried to 950 g kg^-1^ dry matter at ambient temperature. Fish oil and soy lecithin were coated into pellets using a vacuum coater (ZJB-100). Pellet length and diameter were determined using a digital caliper. Durability and breaking point were estimated for uncoated pellets using a pellet tester (ST-136, Shengtai Instrument Co., Ltd., Jinan, China) and hardness tester (ST-120B, Shengtai Instrument Co., Ltd., Jinan, China), respectively. Water stability and sinking rate were determined according to the methods described by Baeverfjord et al. (2006) and Sørensen et al. (2012), respectively.

### 2.3 Fish rearing and experimental conditions

A 72-day feeding trial followed by a 10-day digestibility evaluation experiment were conducted in an indoor recirculation system at the Haid Group research station, Seagull Island, Panyu, Guangzhou, China. Freshwater-acclimated Japanese seabass (*L. japonicus*) juveniles were obtained from a hatchery in Fujian, China and fed a commercial diet for 1 month to adapt to the laboratory condition. Before the experiment, 630 fish with an initial weight of 10.73 ± 0.33 g were fasted for 24 h, anaesthetized with MS-222 (90 mg L^−1^; Hangzhou Dongbao, Hangzhou, China), batch-weighed, and then randomly assigned to 21 circular tanks, with each diet being assigned triplicates and 30 fish per tank. All fish were fed four meals per day by hand (07:30, 10:30, 13:30, and 16:30 h), with each meal lasting 60 min. Fish were fed 10% in excess based on average feed intake over the previous 3 days. All uneaten feed was immediately sieved and dried to a constant weight at 95°C and reweighed. During the feeding experiment, the water temperature ranged from 28.5°C to 30.0°C, dissolved oxygen exceeded 5.0 mg L^−1^, the pH value was 6.5–7.0, ammonia nitrogen and nitrite were less than 0.1 mg L^−1^, and the photoperiod was 12D:12L.

### 2.4 Sampling

Before the experiment, 30 fish were euthanized by an overdose of MS-222 and stored at −20°C for whole-body composition analyses. At the end of the experiment, fish were anaesthetized with 90 mg L^-1^ MS-222 before sampling. Five fish from each tank were individually measured for weight and body length, then dissected to remove the whole viscera; the liver and carcass were weighed separately. Another six fish were randomly sampled from each tank and then kept at −20°C for analysis of whole-body and amino acid compositions. The remaining fish in each tank were assessed for the digestibility evaluation experiment. Faeces were collected by carefully dissecting the abdomen from the last 5 cm of the distal intestine, pooled by tank, and then stored at −20°C.

### 2.5 Chemical analysis

Initial and final whole-body samples from the same tank were cut into pieces and ground with a meat grinder, then autoclaved (YXQ-LS, Xunbo, Shanghai, China) at 120°C for 30 min, homogenized by a homogenizer (DS-1, Shanghai, China), and oven-dried (Jinghong, Zhejiang, China) at 80°C. Whole-body samples were finely ground with a pestle and mortar until all samples passed through a 0.9 mm screen. Dried whole fish and feed samples were analyzed for dry matter (105°C to constant weight), protein (Kjeldahl’s method), lipids (Soxhlet Extraction System, Jingke, Shanghai, China), amino acids (amino acid analyzer, L-8900, Hitachi, Japan), ash (550°C, overnight), and energy (Phillipson Microbomb Calorimeter, Gentry Instruments Inc., Aiken, SC, United States) based on the previous study (Zhang et al., 2022). Faeces were freeze-dried at −50°C (Labonco Freezon 4.5, Kansas City, United States) and ground with a pestle and mortar. Yttrium oxide in feed and faeces was determined based on the previous study using inductively coupled plasma mass spectrometry (ICP-MS) (Zhang et al., 2012a). Briefly, approximately 50 mg of freeze-dried sample was weighed into a digestion tube, and then 6 ml HNO_3_ and 2 ml hydrogen peroxide were added. After microwave digestion, the sample was diluted to a constant volume of 50 ml, aspirated 1 ml diluent, and then re-diluted to 10 ml for on-board testing.

### 2.6 Calculations and statistical analysis

Apparent digestibility (ADAA, %) of individual amino acids was calculated as 100 × (1 - AA_f_ × AA_d_^-1^ × Y_d_ × Y_f_^−1^), where AA_f_ and AA_d_ represent the concentration of individual amino acids in faeces and the diet and Y_f_ and Y_d_ represent the concentration of yttrium in faeces and the diet. Retention of nitrogen and energy (%) was calculated as 100 × (N_1_ × FBW - N_0_ × IBW) × (N_d_ × FI)^−1^, where N_0_ and N_1_ represent the nutrient or energy in the initial and final whole body and N_d_ represents the nutrient or energy in the diet. Feed intake (FI, %/d) was quantified by subtracting uneaten feed from the amount of feed on a dry matter basis. The feed conversion ratio (FCR) was calculated as FI × (weight gain, g)^−1^. The weight gain rate (WGR, %) was calculated as 100 × (FBW–IBW)/IBW, where FBW and IBW represent final body weight and initial body weight, respectively. The hepatosomatic and viscerosomatic indices (%) were expressed as organ weight as a percentage of body weight, and the condition factor (CF, g/cm^3^) was calculated as 100 × (fish weight, g) × (body length, cm)^−3^.

All data are presented as means ± pooled S.E.M. (n = 3) and assessed by one-way ANOVA (IBM, SPSS Statistics 20.0), with the level of significance set at *p* < 0.05. Moreover, a follow-up trend analysis using orthogonal polynomial contrasts was performed to determine whether the significant effects were linear and/or quadratic. Quadratic regressions were only presented when the regression coefficient of the second degree component was statistically significant (*p* < 0.05). Maxima or minima in quadratic regressions were calculated by setting the first derivative of the equation to 0. Significant differences in the ANOVA were ranked by the Pdiff routine under LSMEANS and indicated by different superscript letters ^a, b, c^.

## 3 Results

### 3.1 Extrusion parameter and physical pellet quality

The processing parameter and pellet physical quality are presented in Table 5. The V0 diet differed from the other diets, and the total amount of process water was limited to 20% to achieve a uniform flow through the extruder. The combined effect of dietary composition and restricted water addition resulted in a lower bulk density, breaking point, and slower sinking rate compared with the other diets. The diametric expansion of all diets ranged from 74% to 88% and increased with increasing dietary wheat gluten. All diets had similar durability, with VS1 being the most water-stable and VF1 the least.

**TABLE 5:** Extrusion processing parameters and feed pellet physical quality.

Diet	V0	VF1	VF2	VF3	VS1	VS2	VS3
*Extruder parameters*							
Feeding rate, kg h^-1^	125	125	125	125	125	125	125
Water addition in conditioner, %	8	12	16	16	14	16	16
Water addition in extruder, %	8	8	12	12	12	12	12
Die temperature, **°**C	117	87	86	86	88	85	85
Revolution screws, rpm	259	259	280	280	280	280	280
Cutter speed, rpm	2,300	2,400	2,350	2,350	2,400	2,450	2,400
Physical quality							
Length, mm	4.63	3.98	4.31	4.34	3.93	3.77	3.83
Diameter, mm	3.50	3.48	3.59	3.76	3.53	3.57	3.67
Expansion, %	74.9	74.1	79.3	87.9	76.7	78.7	83.4
Bulk density, g L^-1^	518	561	571	563	561	561	550
Breaking point, N	24.7	37.1	35.0	34.7	32.4	34.0	35.4
Water stability, %	88.8	79.7	86.0	84.7	89.1	86.4	85.1
Sinking rate, cm s^-1^	7.94	9.03	8.37	8.24	9.26	9.78	9.47

### 3.2 Growth performance and feed utilization

No significant differences were found in FI (Table 6). WGR linearly and significantly decreased from 795% to 627% with increasing dietary GWT. Nitrogen retention significantly decreased from 34.8% to 26.9%, while energy retention decreased from 40.2% to 32.5%. FCR linearly increased from 1.07 to 1.36 as GWT gradually replaced FM. No significant differences were found in WGR or FCR with GWT gradually replacing SPC. The retention of nitrogen and energy linearly decreased with the increase in GWT supplementation.

**TABLE 6:** Growth performance and feed utilization of Japanese seabass fed different experimental diets.

Parameters	Diets				Pooled S.E.M^a^	*P*	Regression model	R^2^
	V0	VF1	VF2	VF3				
Feed intake (FI), g DM fish^-1^	86.0	81.1	83.1	85.9	6.16	0.65	—	—
Weight gain (WGR), %	795^a^	725^ab^	646^b^	627^b^	63.14	0.019	786–1.75 x	0.66
Specific growth rate (SGR), %/d	3.04^a^	2.93^ab^	2.79^b^	2.75^b^	0.118	0.027	3.03–3.06 * 10–3 x	0.63
Feed conversion ratio (FCR), g FI (g WG)^−1^	1.07^b^	1.11^b^	1.28^a^	1.36^a^	0.065	<0.001	1.05 + 3.08 * 10^−3^ x	0.83
Nitrogen retention, %	34.8^a^	33.6^a^	29.2^b^	26.9^c^	1.28	<0.001	35.3–0.0843 x	0.89
Energy retention, %	40.2^a^	38.0^a^	33.4^b^	32.5^b^	2.46	<0.01	40.2–0.0837 x	0.73
	**V0**	**VS1**	**VS2**	**VS3**				
FI, g DM fish^-1^	86.0	85.3	84.9	87.5	5.78	0.92	—	—
WGR, %	795	757	747	766	56.82	0.67	—	—
SGR, %/d	3.04	2.98	2.97	3.00	0.091	0.67	—	—
FCR, g FI (g WG)^−1^	1.07	1.12	1.13	1.13	0.051	0.39	—	—
Nitrogen retention, %	34.8^a^	32.5^b^	32.2^b^	31.9^b^	0.90	<0.01	34.7–0.0723 x + 4.56 * 10^−4^ x^2^	0.73
Energy retention, %	40.2^a^	37.4^b^	37.0^b^	37.4^b^	1.02	<0.01	40.2–0.0977 x + 7.07 * 10^−4^ x^2^	0.75

Pooled standard error of means. Data are means and pooled S.E.M. different superscript letters ^a^ ^,b,^ and^c^ indicate significant (*p* < 0.05) differences among treatments.

### 3.3 Whole-body composition and somatic index

Moisture, protein, lipid, ash, gross energy in the whole body, and the hepatosomatic index and condition factor were not significantly affected by different dietary GWT supplementation (Tables 7, 8). The viscerosomatic index linearly decreased from 17.3% to 16.1% with the increase of GWT supplementation.

**TABLE 7:** Morphologic index of Japanese seabass fed different experimental diets.

Somatic indices	Diets				Pooled S.E.M.	*P*	Regression model	R^2^
	V0	VF1	VF2	VF3				
Hepatosomatic index (HSI), %	1.52	1.66	1.55	1.38	0.263	0.57	—	—
Viscerosomatic index (VSI), %	17.3	17.3	17.8	17.5	0.64	0.64	—	—
Condition factor (CF), g cm^-3^	1.78	1.79	1.70	1.66	0.116	0.36	—	—
	**V0**	**VS1**	**VS2**	**VS3**				
HSI, %	1.52	1.51	1.57	1.36	0.348	0.84	—	—
VSI, %	17.3^a^	17.0^a^	16.6^ab^	16.1^b^	0.48	0.031	17.4–0.0125 x	0.64
CF, g cm^-3^	1.78	1.74	1.79	1.74	0.069	0.69	—	—

Data are means and pooled S.E.M. different superscript letters ^a^ and ^b^ indicate significant (*P* 0.05) differences among treatments.

**TABLE 8:** Whole-body compositions of Japanese seabass fed different experimental diets.

Whole-body composition, kg^-1^	Diets				Pooled S.E.M.	*P*
	V0	VF1	VF2	VF3		
Moisture, g	659	664	664	655	8.9	0.44
Crude protein, g	164	166	164	163	3.4	0.68
Crude fat, g	123	119	119	131	7.2	0.16
Ash, g	44.0	43.4	42.2	41.5	1.36	0.11
Gross energy, MJ	8.95	8.78	8.83	9.23	0.307	0.23
	**V0**	**VS1**	**VS2**	**VS3**		
Moisture, g	659	670	666	664	12.4	0.69
Crude protein, g	164	161	163	164	5.8	0.92
Crude fat, g	123	118	117	123	8.0	0.66
Ash, g	44.0	42.1	42.5	41.2	1.90	0.30
Gross energy, MJ	8.95	8.67	8.74	8.90	0.378	0.69

Data are means and pooled S.E.M.

### 3.4 Apparent digestibility coefficient (ADC) of amino acids

No significant differences were found in the ADCs of Arg, Ile, Met, Thr, Tyr, and Val (Table 9). As GWT gradually replaced FM, the ADC of Cys significantly increased from 77.6% to 86.5%. The ADCs of His, Lys, and total AAs followed a similar pattern and increased with the increase in GWT supplementation. The ADCs of Leu, Phe, and total EAAs increased in a curvilinear mode with increasing proportions of GWT. Except for Cys and Met, the ADCs of essential and total amino acids were not significantly affected by the gradual substitution of SPC by GWT. The ADC of Cys significantly increased from 77.6% to 90.0%, while the ADC of Met significantly increased from 66.8% to 76.7%. The ADCs of Cys and Met were lower in the V0 diet than in the other diets.

**TABLE 9:** Apparent digestibility coefficient (ADC) of amino acids of Japanese seabass fed different experimental diets.

ADC, %	Diets				Pooled S.E.M.	*P*	Regression model	R^2^
	V0	VF1	VF2	VF3				
Arg	93.7	94.2	95.2	94.3	1.52	0.60	—	—
Cys	77.6^b^	81.7^ab^	84.8^a^	86.5^a^	3.42	0.027	78.2 + 0.0893 x	0.64
His	90.2^b^	92.0^ab^	93.3^a^	93.5^a^	1.45	0.042	90.6 + 0.0337 x	0.57
Ile	84.5	89.2	89.8	88.3	2.36	0.053	—	—
Leu	87.0^b^	91.4^a^	92.0^a^	90.9^a^	2.08	0.036	87.1 + 0.160 x - 1.23 * 10^−3^ x^2^	0.63
Lys	93.2^b^	94.3^ab^	95.0^a^	94.9^a^	0.68	0.020	93.5 + 0.0175 x	0.57
Met	66.8	71.6	70.3	69.3	3.42	0.30	—	—
Phe	89.1^b^	92.5^a^	93.2^a^	92.8^a^	1.52	0.017	89.2 + 0.121 x - 8.60 * 10^−4^ x^2^	0.69
Thr	82.4	86.8	87.7	87.0	2.46	0.055	—	—
Tyr	86.0	90.1	91.0	89.4	2.21	0.057	—	—
Val	84.5	89.1	89.6	88.3	2.45	0.069	—	—
EAAs	87.4^b^	90.5^a^	91.3^a^	90.5^a^	1.67	0.041	87.4 + 0.120 x - 8.93 * 10^−4^ x^2^	0.62
Total AAs	87.3^b^	90.4^a^	91.5^a^	91.4^a^	1.70	0.023	88.1 + 0.0408 x	0.52
	**V0**	**VS1**	**VS2**	**VS3**				
Arg	93.7	95.0	94.2	95.5	1.63	0.44	—	—
Cys	77.6^b^	87.7^a^	86.2^a^	90.0^a^	2.90	0.001	80.0 + 0.107 x	0.61
His	90.2	92.7	91.5	92.8	2.01	0.29	—	—
Ile	84.5	90.4	87.7	88.5	4.65	0.41	—	—
Leu	87.0	92.2	89.9	91.4	3.82	0.30	—	—
Lys	93.2	94.9	94.9	95.8	1.09	0.054	—	—
Met	66.8^b^	74.7^a^	73.2^a^	76.7^a^	3.85	0.031	68.6 + 0.0843 x	0.47
Phe	89.1	93.1	91.3	92.8	2.69	0.22	—	—
Thr	82.4	88.7	86.4	88.8	3.80	0.13	—	—
Tyr	86.0	91.1	88.3	89.9	3.93	0.35	—	—
Val	84.5	90.4	87.4	89.3	4.24	0.29	—	—
EAAs	87.4	91.6	89.8	91.4	2.88	0.22	—	—
Total AAs	87.3	91.6	90.1	92.1	2.49	0.092	—	—

Data are means and pooled S.E.M. different superscript letters ^a^ and^b^ indicate significant (*p* < 0.05) differences among treatments.

### 3.5 Retention of essential amino acids

The retention of individual and total EAAs significantly decreased in a linear manner (Table 10). The retention of Met was twice or more than the values observed in the other EAAs. The replacement of SPC with GWT had no significant effect on the retention of Arg, His, Ile, Leu, Lys, Phe, Thr, Val, and total EAAs. The retention of Met followed a U-shaped pattern, with the highest value being found in the V0 diet.

**TABLE 10:** Retention of essential amino acids (EAAs) of Japanese seabass fed different experimental diets.

Retention of digestible EAAs, %	Diets				Pooled S.E.M.	*P*	Regression model	R^2^
	V0	VF1	VF2	VF3				
Arg	31.7^a^	31.7^a^	27.0^b^	25.4^b^	1.31	<0.001	32.5–0.0712 x	0.80
His	31.9^a^	32.5^ab^	26.2^bc^	24.4^c^	1.30	<0.001	33.1–0.0866 x	0.79
Ile	35.0^a^	34.1^ab^	30.3^b^	27.9^b^	1.36	<0.001	35.6–0.0760 x	0.86
Leu	35.1^a^	33.7^ab^	29.2^bc^	27.0^c^	1.32	<0.001	35.6–0.0868 x	0.89
Lys	41.5^a^	42.1^a^	35.2^b^	33.5^ab^	1.60	<0.001	42.7–0.0919 x	0.77
Met	86.7^a^	81.2^ab^	77.6^bc^	71.4^c^	4.08	<0.01	86.6–0.148 x	0.78
Phe	31.4^a^	30.0^ab^	24.1^bc^	22.4^c^	1.13	<0.001	31.9–0.0988 x	0.89
Thr	42.2^a^	40.6^ab^	35.1^b^	32.8^b^	1.71	<0.001	42.7–0.101 x	0.87
Val	36.3^a^	35.5^ab^	31.5^bc^	29.6^c^	1.47	<0.001	36.8–0.0716 x	0.83
EAA	37.2^a^	36.5^ab^	31.2^bc^	29.1^c^	1.40	<0.001	37.9–0.0887 x	0.86
	**V0**	**VS1**	**VS2**	**VS3**				
Arg	31.7	29.6	30.3	29.8	1.12	0.11	—	—
His	31.9	31.0	32.8	33.5	1.31	0.11	—	—
Ile	35.0	32.3	34.5	35.5	2.39	0.32	—	—
Leu	35.1	32.1	33.8	33.8	1.89	0.25	—	—
Lys	41.5	39.2	40.9	40.7	1.31	0.17	—	—
Met	86.7^a^	66.3^b^	66.0^b^	60.5^b^	4.39	<0.001	85.4–0.572 x + 3.35 * 10^−3^ x^2^	0.84
Phe	31.4	28.9	30.0	29.3	1.34	0.12	—	—
Thr	42.2	37.6	39.4	38.6	2.22	0.084	—	—
Val	36.3	32.6	35.0	35.3	2.15	0.18	—	—
EAA	37.2	34.1	35.7	35.5	1.67	0.16	—	—

Data are means and pooled S.E.M. different superscript letters ^a,b^, and^c^.indicate significant (*p* < 0.05) differences among treatments.

## 4 Discussion

The bulk density of the pellets was optimized by adjusting the amount of water in the preconditioner and extruder so that they slowly sunk into the water (Zhang et al., 2012b). A minimum amount of water (16%) was added during the extrusion of diet V0 (control diet). Diet VF1 had 20% water supplementation and the other diets had 26%–28% water supplementation. The low water supplementation during the extrusion of the V0 diet reduced the bulk density, sink rate, and breaking force of the pellets, which was different from the VF1 diet. The increase in dietary GWT resulted in greater diametric expansion due to the viscoelastic nature of wheat gluten. This is in accordance with previous findings reported by Draganovic et al. (2011), who found that an increased proportion of wheat gluten to soy protein concentrate led to increased dietary hardness. Additionally, wheat gluten affects extrusion characteristics and physical qualities, including pellet hardness and durability (Day et al., 2006; Wieser, 2007; Apper-Bossard et al., 2013). However, an opposite trend of hardness was observed in diets VF1, VF2, and VF3 when GWT replaced FM, which may be associated with the degree of expansion and water addition (Sørensen et al., 2012; Storebakken et al., 2015b).

In this study, the SGR and FCR of fish fed the high fishmeal diet (V0 diet) were 3.04%/d and 1.10. This growth performance is similar to the previous growth responses (SGR = 3.52), with 52% fish meal-based diets being fed to comparably sized juvenile Japanese seabass (Cheng et al., 2010). Similarly, a study by Xu et al. (2010) reported that Japanese seabass fed a diet with 45% fish meal showed the SGR and FCR were 2.84%/d and 0.99, respectively. Studies have shown that a diet containing 41% protein and 12% lipids is optimal for Japanese seabass juveniles with an initial body weight (IBW) of 6.26 g (Ai et al., 2004). The current results showed that the growth performance of seabass fed 20% fish meal was similar to those fed 40% fish meal, which illustrated the potential of using plant protein in Japanese seabass. This is in keeping with studies in European sea bass (*Dicentrarchus labrax*) and gilthead seabream (*Sparus aurata* L.) reported by Messina et al. (2013); Kissil and Lupatsch (2004), respectively. The lack of significant feeding stimulation in high GWT supplementation suggests that the incorporation of 5% krill meal is needed to provide sufficient marine attractant to ensure the high feeding intake of Japanese seabass. This result is consistent with previous observations in Atlantic salmon (Storebakken et al., 2000a), Atlantic halibut (Helland and Grisdale-Helland, 2006), rainbow trout (Storebakken et al., 2015a), and Asian seabass (Apper et al., 2016), suggesting that small amounts of marine components are required to ensure high feed intake when providing a high level of wheat gluten.

Despite rapid growth and high feed conversion, Japanese seabass fed the control diet showed signs of essential amino acid deficiency. The same situation was also found in fish that were fed diets in which fish meal and SPC were gradually replaced with GWT. The availability of Met by growth mainly depends on digestibility, bioactivity, and catabolism. Low water supplementation during extrusion of the V0 diet resulted in lower digestibility and bioactivity of sulfur-containing amino acids, which led to lower digestibility of Met (66.8%) and Cys (77.6%) in the V0 diet. The digestibility of amino acids in wheat gluten was higher than in LT fish meal, especially Cys, which is consistent with previous findings in Atlantic salmon (Storebakken et al., 2000a). Dietary concentrations of Cys and Met are often reported together mainly due to the fact that sufficient Cys stimulates the downregulation of Met catabolism. However, no significant difference was found in the regression of digestible Cys and Met on nitrogen retention (R_2_ = 0.43) in this study. The ratio of digestible Met and Cys (DMDC) was linearly correlated with protein retention (Ret_N_ = 13.6 + 14.6 * DMDC, R_2_ = 0.93). This linear relationship can be expressed by replacing GWT with fish meal (DMDC = 1.49–1.17 * 10^−2^ * Rep, R_2_ = 0.973). Regression analyses showed that the highest nitrogen retention was obtained in the diets with a low replacement of protein from FM with GWT. Replacement of SPC with GWT also resulted in increased concentrations and digestibility of Cys and Met. Additionally, the retention of digestible methionine decreased with increasing GWT supplementation, indicating methionine deficiency.

## 5 Conclusion

The results of the present study indicated that replacing protein in fish meal with wheat gluten reduced growth, feed efficiency, and the retention of nitrogen, energy, and essential amino acids. Replacement protein in soy protein concentrate with wheat gluten had no significant effects on feed intake, growth, and the feed conversion ratio, but resulted in a lower retention of nitrogen, energy, and Met. Moreover, wheat gluten increased amino acid digestibility compared with soy protein concentrate and fish meal. This study highlights that a moderate amount of wheat gluten is a promising dietary protein alternative for Japanese seabass.

## Data Availability

The original contributions presented in the study are included in the article/supplementary material, further inquiries can be directed to the corresponding author.
